# A multi-label visualisation approach for malware behaviour analysis

**DOI:** 10.1038/s41598-025-21848-z

**Published:** 2025-10-30

**Authors:** Dilara T. Uysal, Paul D. Yoo, Kamal Taha, Chan Yeob Yeun, Ernesto Damiani

**Affiliations:** 1 Birkbeck College, University of London, London, WC1E 7HX UK; 2 Center for Cyber-Physical Systems (C2PS), Khalifa University of Science and Technology, Abu Dhabi, UAE; 3 Department of Computer Science, University of Milan, Milan, Italy

**Keywords:** Malware Detection, Object Detection, Explainability, Computer science, Information technology

## Abstract

Modern malware evolves continuously, posing persistent challenges to cybersecurity. Conventional classification approaches typically group malware by its primary objective, emphasising dominant behaviours while overlooking the complex and overlapping strategies common in real-world attacks. Here we present DECODE (DEep Classification Of Dynamic Exploits), a proportional multi-label, context-aware framework that combines object detection, explainable artificial intelligence (XAI), and agent-based large language models (LLMs) to deliver interpretable and comprehensive malware analysis. DECODE introduces the first object detection dataset specifically for malware classification, generated through an automated annotation pipeline that removes the need for manual labelling and remains effective even for visually indistinguishable malware features. To improve attribution reliability, we extend Gradient-weighted Class Activation Mapping (Grad-CAM) with a Bayesian formulation, enabling uncertainty-aware visualisation of discriminative regions linked to multiple categories. The regions identified through object detection are subsequently mapped to their corresponding API call sequences and interpreted via a multi-agent reasoning module, which incorporates critique-and-verification loops to reduce hallucinations and bias. Experimental evaluation shows multi-label and binary classification accuracies of 0.8513 and 0.9380, respectively, outperforming conventional deep learning baselines. By combining visual localisation, proportional multi-label scoring, and human-readable behavioural narratives, DECODE enables malware to be classified not only by intended impact but also by fine-grained structural and behavioural traits, offering a richer understanding of complex threats.

## Introduction

Deep learning has significantly advanced the detection and classification of malware variants, offering superior performance over traditional methods^1^. However, its decision-making processes remain opaque, creating a “black box” problem that limits trust and interpretability. Explainable AI (XAI) addresses this challenge by clarifying how black-box models operate and how predictions are derived^2^, a capability of particular importance in cybersecurity, where understanding the rationale behind classifications is essential^3^.

Existing interpretable malware classification methods, however, primarily focus to explain individual decisions and rarely rarely capture the compositional structure of malware, particularly the proportional contributions of overlapping behavioural categories. Modern malware often exhibits complex, multi-faceted behaviours that cannot be adequately represented by a single dominant category. Restricting interpretation to the most salient behaviour risks oversimplifying a sample’s functional scope and obscuring latent, yet operationally relevant, capabilities. For example, WannaCrypt (also known as WanaCrypt0r, WCRY, WanaDecrypt0r, WCrypt) is classified as ransomware yet demonstrates worm-like propagation, self-replicating across networks while encrypting files and demanding ransom^4^. Such cases emphasise the need for frameworks that account for behavioural overlap rather than enforcing single-category assignments.

Several works have explored more granular or behaviourally aware classification paradigms. MAEC^5^ standardises the characterisation and exchange of malware behaviours across tools and organisations, while Yavvari et al.^6^ model malware as modular behavioural units, recognising that a single sample may exhibit multiple behaviours. Trizna et al.^7^ employ knowledge graph embeddings to represent malware entities and their relationships in an *n*-dimensional space, enabling interpretable characterisation through structured relationships and description logic-based explanations. Li and Fung^8^ build functional profiles from API call sequences, map them to assembly-level function clusters, and quantify each cluster’s contribution, providing fine-grained interpretability. However, none of these approaches quantify the proportional contributions of overlapping categories within a sample or provide direct, visually grounded feature attribution.

To address these gaps, we introduce DECODE, a proportional multi-label classification framework that quantifies the percentage representation of multiple malware categories within a single sample. By integrating multi-label classification with visual explanation techniques, DECODE enables classification not only by dominant effects but also through a comprehensive examination of hybrid and overlapping traits, thereby enhancing interpretability and operational insight. Behavioural features are transformed into image-based representations, and object-level feature detection is applied to localise discriminative regions and associate them with specific categories. An automated annotation pipeline extends Grad-CAM with a Bayesian formulation to produce uncertainty-aware and reliable discriminative regions from behaviour-derived images, ensuring that only the most informative features are selected. These curated annotations form a high-quality object detection dataset used to train the multi-label classification model. For previously unseen samples, detected regions are mapped back to their corresponding API call sequences and interpreted using a multi-agent reasoning module, generating human-readable and verifiable behavioural narratives. Our findings show that malware samples often display characteristics from multiple categories simultaneously, highlighting the complexity of modern threats and the limitations of single-category classification.

Contributions of this work are:

1. We introduce the first object detection dataset for malware classification, generated through an automated annotation pipeline capable of localising discriminative features even when visually indistinguishable.
2. We propose a Bayesian extension to Grad-CAM to enable uncertainty-aware feature attribution, enhancing interpretability and reliability.
3. We develop a proportional multi-label scoring mechanism that attributes distinct behavioural feature regions to multiple categories, providing a richer representation of hybrid and overlapping behaviours.

The rest of this paper is organised as follows. Section II presents a new concept of ‘explainability through visualisation’ using a multi-labelling approach, addressing existing gaps in the literature and discussing the construction of the framework. Section III summarises the experimental results obtained in the research, emphasising the enhancement of transparency in the decision-making process. Section IV provides a brief conclusion and suggests directions for future research.

## Method

Standardising malware characterisation through behaviours, artifacts, and attack patterns accurately captures how malware operates and the actions it performs. This approach enhances detection capabilities while also aiding in the assessment of malware objectives and potential risks^5^. Key feature extraction and identification form the first and most crucial step of our study to uncover the “DNA” of malware.

**Fig. 1:**
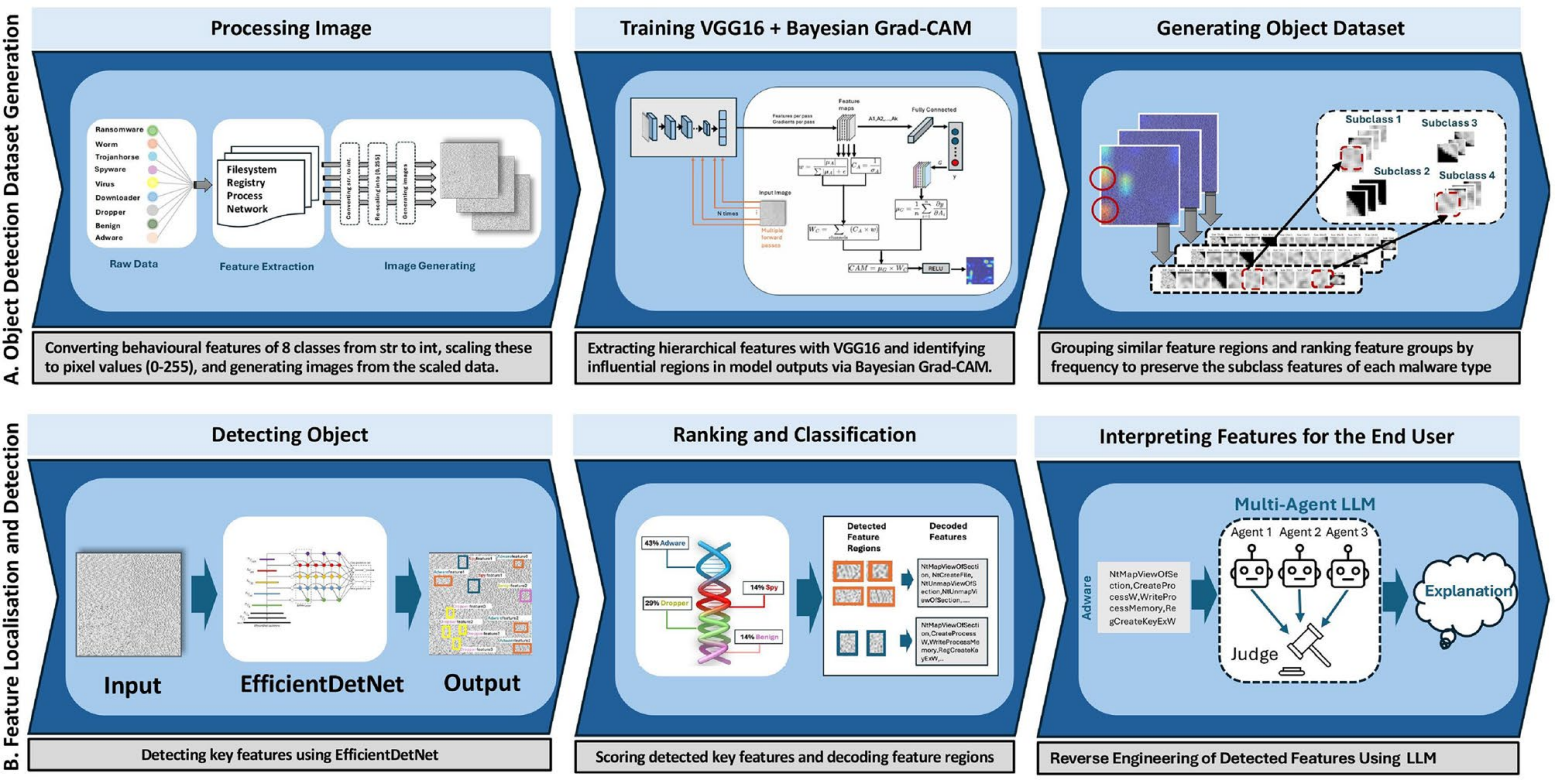
Two-Stage Framework for Malware Analysis. Step A - Feature Extraction and Identification: raw malware behavioural data is transformed into image representations, and Bayesian Grad-CAM is applied to highlight key discriminative regions across malware types. These highlighted regions are then grouped according to visual and behavioural similarity to construct an object detection dataset that captures category-specific patterns. Step B - Feature Localisation and Detection: the identified regions are isolated for object detection and classification. The detected visual features are mapped back to their corresponding API calls and explained using a multi-agent LLM framework to enhance interpretability and provide human-readable reasoning.

The proposed framework comprises several components essential for exploring the potential of explainability in the malware detection process. As shown in Fig. 1, the framework is divided into two stages: **feature extraction and identification** in the first stage, followed by **feature localisation and detection** in the second stage. The architectural design integrates multiple critical elements into a unified system. It involves generating images from raw malware data, identifying features, creating datasets based on the extracted features, and uncovering key characteristics through detection and classification. To further enhance interpretability, the framework incorporates **a multi-agent module**. This module translates the detected feature regions back into their corresponding API call names utilising a newly developed multi-agent module, consisting of Reviewer, Adversarial, Consensus, and Verifier agents, to generate human-readable explanations. These explanations provide insights into the intent and functional significance of each detected feature, grounded in domain-specific knowledge, the MITRE ATT&CK framework. This step ensures that the entire detection process is not only accurate but also transparent and explainable to analysts and end users.

### Key feature extraction and identification

This section outlines the dataset generation process for the object detection task, which constitutes the first stage of the DECODE framework. The pipeline includes multiple stages: behavioural feature extraction, malware image generation, discriminative region identification using a Bayesian extension of Grad-CAM, and the grouping and scoring of key feature regions by malware category. Together, these steps constitute an automated pipeline for constructing a malware object detection dataset. Emphasis is placed on achieving visual interpretability of malware behaviours and supporting a multi-label classification framework that reflects the complex, overlapping nature of real-world malware threats.

#### Dataset

Static analysis examines binary files without execution, extracting structural features such as strings and headers to infer malware intent. It is fast and computationally efficient but remains vulnerable to obfuscation techniques. In contrast, dynamic analysis involves executing malware within a controlled virtual environment to observe its behaviour in real time. Unlike static methods, dynamic analysis offers greater robustness against obfuscation^9^ and provides valuable insights into the operational goals and behaviours of malware. These methods often leverage the frequency and sequence of API calls, as such patterns reveal distinctive behavioural characteristics critical for detecting malicious activity. The frequency of API calls indicates their importance to the malware’s functionality, while the sequence provides details about its step-by-step actions. API functions, integral components of the operating system, facilitate access to essential system resources, such as file systems, processes, and the Windows registry^10^.

Kakisim et al.^10^ evaluate the effectiveness of various malware detection strategies by analysing dynamic features, including API calls, interactions with system libraries, and manipulations of mutexes, registries, and files. Their study highlights the API-Bigram approach, which combines frequency analysis with sequential context, as a powerful method for distinguishing malware families and identifying suspicious programs. The findings demonstrate that both the API-Bigram and API-Frequency approaches deliver superior results for classifying malware families across diverse datasets. Building on this foundation, the next step involves selecting features that can effectively distinguish between malware categories. By leveraging the behavioural patterns captured during dynamic analysis, we identify key characteristics that highlight the distinct nature of each category.

Image-based malware data provide a more efficient method for managing and analysing malware behaviour, as instances of the same type often display recognisable and consistent patterns. To address the absence of publicly available dataset for malware dynamic feature images, we developed our own image-based dataset. We collected samples from eight categories: worm, ransomware, spyware, dropper, adware, downloader, virus, and benign, with approximately 1,000 samples per category. To ensure a balanced and diverse dataset, malware samples were sourced from MalwareBazaar^11^, VirusShare^12^, and VirusTotal^13^, all of which are regularly updated repositories. MalwareBazaar and VirusTotal provide labelled datasets that support family-based sampling. To ensure the accuracy of labels from MalwareBazaar and to assign labels to samples from VirusShare, we used the VirusTotal API. Each malware sample was labelled according to the most frequently detected malware type across multiple antivirus engines. For benign applications, we downloaded executable files from a curated list of popular Windows programs^14^ and confirmed their benign status using the VirusTotal API. Files that produced no alerts from any antivirus engine were classified as benign.

Executable samples were analysed using CAPE Sandbox to extract network, registry, file system, and process features, enabling detailed examination of behavioural patterns. In DECODE, we convert the extracted features into an image with pixel values ranging from 0 to 255, mapping each string in the report to an integer. This representation facilitates the identification of unique patterns associated with different malware categories. It is important to note that certain API names are deliberately duplicated by malware authors to mislead analysts and obscure behavioural patterns. To mitigate this, consecutive duplicate API calls are filtered, retaining only a single instance of each repeated entry. For each feature type, a fixed image size of 128$\times$128 pixels (16,384 pixels in total) is employed. This resolution was selected to balance semantic richness and computational efficiency. On average, malware samples contained approximately 20,000 API calls per feature type. To fully populate the image frame, sequence elements are repeated as necessary; however, to minimise redundancy and maintain structural integrity, the image size per feature type is strictly capped at 16,384 pixels. This ensured compatibility with CNN-based models and avoided distortions that can occur from arbitrary resizing.

When a sequence exceeded this threshold, an iterative cleaning procedure was applied to reduce redundancy across all behavioural feature types. This process includes up to 20 refinement steps and employs two primary strategies: (1) the iterative removal of consecutive duplicate API calls using a fixed group size that increases with each iteration, and (2) a sliding window-based approach that removes variable-size repeated groups. The sliding window is initially configured with a size of 10 and expands incrementally up to half the sequence length, enabling detection and elimination of recurring API call groups. To evaluate the impact of this cleaning process, three dataset variants were prepared:

1. baseline without repeated-group cleaning,
2. sliding-window minimum size = 5, and
3. sliding-window minimum size = 10.

Following group-level cleaning, a final pass eliminated any residual consecutive duplicates. The refinement continued until the sequence length was reduced to at most 16,384 elements. If the number of API calls remained below this threshold, existing entries were duplicated sequentially from top to bottom until the fixed frame was fully populated. This procedure ensured that all samples were represented with a consistent image layout while retaining the most informative API calls for downstream analysis.

To upscale the final 128$\times$128 images to 256$\times$256 resolution, we employed the Resampling.NEAREST method from the Pillow library. This technique duplicates pixel values without interpolation, thereby preserving the structural integrity of individual pixels. In contrast, the Resampling.LANCZOS method introduced noticeable blurring and distortion during upsampling. As a result, Resampling.NEAREST was selected to maintain consistent and undistorted feature representation.

Each set of four distinct features corresponds to a specific region within the image, which is then divided into frames (256, 256). These frames are allocated to individual features for analysis or processing. For instance, in an image of one instance (512, 512), the initial frame coordinates (0, 256, 0, 256) pertain to the network feature.

Since many malware samples exhibit shared or similar behaviours, some of the initially generated images are found to be identical. To address this, we implemented a cleaning process to remove such duplicated images and increase the diversity of the dataset. This process involves iterative removal of fixed-size duplicate groups (e.g., 2, 3, or 4 consecutive API names). For example, we first remove duplicate groups of size 2. If identical images are still detected after this step, we continue the cleaning process by removing repeating groups of size 3, and so on. This progressive deduplication strategy helps generate more distinct images, thereby reducing redundancy in the training set and mitigating the risk of overfitting during model training.

Initially, each category consisted of around 1,000 samples: adware (903), virus (905), ransomware (895), worm (928), spyware (904), dropper (1,015), downloader (1,009), and benign (956). Following the data cleaning procedure, which involved removing duplicates and retaining only distinct images, the sample distribution changed to: adware (548), virus (846), ransomware (844), worm (784), spyware (837), dropper (647), downloader (890), and benign (953).

The substantial decrease in unique samples within the adware and dropper categories indicates that their behaviours are highly repetitive, resulting in frequent duplication across images. Conversely, the benign category displayed considerable behavioural diversity, with only three duplicates identified, each corresponding to an identical behavioural pattern.

#### Feature grouping and ranking

Each type of malware exhibits unique characteristics based on its primary objectives. By identifying these specific features, we can extract hereditary traits unique to each malware type. This allows us to analyse malware images to uncover their “DNA,” revealing which features are derived from which types of malware. To identify these features, we trained a classification model and utilised our modified Bayesian Grad-CAM to highlight the regions that most influence the model’s predictions for each specific class. The similarity between highlighted regions is analysed to cluster similar features, which are then ranked to identify the most commonly used features for each specific type of malware.

Our dataset, derived from malware behavioural data, is pattern-based and contains complex features. For feature learning, we trained several architectures, including VGGNet, InceptionV4, ResNet101, ResNet50, and ResNet34 due to their proven effectiveness in a wide range of deep learning tasks in malware detection, to identify the model with the best classification performance for learning complex patterns and features from our dataset.

VGGNet is particularly valued for its simplicity and depth, making it highly efficient for feature extraction with a systematic approach to learning hierarchical features. ResNet, on the other hand, addresses the vanishing gradient problem by incorporating residual connections, which enables the training of deeper networks. We enhanced the ResNet models with attention modules, SENet^15^, and CBAM^16^, which selectively focus on informative features while disregarding irrelevant ones. Lastly, InceptionNet is recognised for its capacity to capture multi-scale features through its inception modules, making it ideal for tasks involving varying feature sizes.

Following comparative evaluation of several architectures, VGG16 was selected as the most effective baseline. Its architecture was customised by inserting dropout layers after the 4th, 9th, 16th, and 23rd layers to enable Bayesian approximation via Monte Carlo dropout during Bayesian Grad-CAM analysis.

To enhance the discriminative capability of the learned features, we implemented a joint loss function in VGG16 as proposed by Wen et al.^17^, combining softmax loss and center loss supervision, as shown in (1) and (2). This joint supervision approach amplifies inter-class feature differences while minimising intra-class feature variations, enabling more effective identification of subtle intra-class features. The following Equations (1) and (2) represent the joint supervision formula. The loss function is defined as:

1 $$\begin{aligned} L = L_S + \lambda L_C \end{aligned}$$

Expanding the terms:

2 $$\begin{aligned} L = -\sum _{i=1}^m \log \frac{e^{W_{y_i}^T x_i + b_{y_i}}}{\sum _{j=1}^n e^{W_j^T x_i + b_j}} + \frac{\lambda }{2} \sum _{i=1}^m \Vert x_i - c_{y_i}\Vert _2^2 \end{aligned}$$

Building upon these refined feature representations, we next create an object detection dataset by detecting and segmenting category-specific behavioural features in the images for each category. Due to the overlapping nature of malware behaviours, it is important to identify distinctive and key features of each malware type that differentiate them from other classes. Techniques such as Grad-CAM [6] produce activation maps that emphasise parts of the image critically affecting the model’s decision-making process. By focusing on features that positively influence class prediction and disregarding negative features as irrelevant to the class, we gain valuable insight into the most impactful features.

Qiu et al.^18^ applied Grad-CAM to a range of deep learning models and observed some differences in the resulting heatmaps across different architectures. They suggested that these variations are likely influenced by the underlying architecture, such as pure convolutional networks, residual networks, or transformer based models as well as differences in network size and depth. Chattopadhyay et al.^19^ also observed that Grad-CAM frequently encounters difficulties in localising multiple instances of the same class within a single image. Due to the repetitive and structurally complex patterns present in malware images, Grad-CAM may likewise struggle to accurately identify and localise key feature regions, which could limit its effectiveness in malware analysis applications.

Due to model uncertainty and instability, as well as the presence of noisy behavioural data with repeated patterns, further refinement is required to generalise its applicability beyond image-based architectures while maintaining meaningful interpretability. To address the instability of Grad-CAM in such contexts, we propose a Bayesian extension that incorporates uncertainty by performing multiple stochastic forward passes using Monte Carlo dropout.

**Fig. 2:**
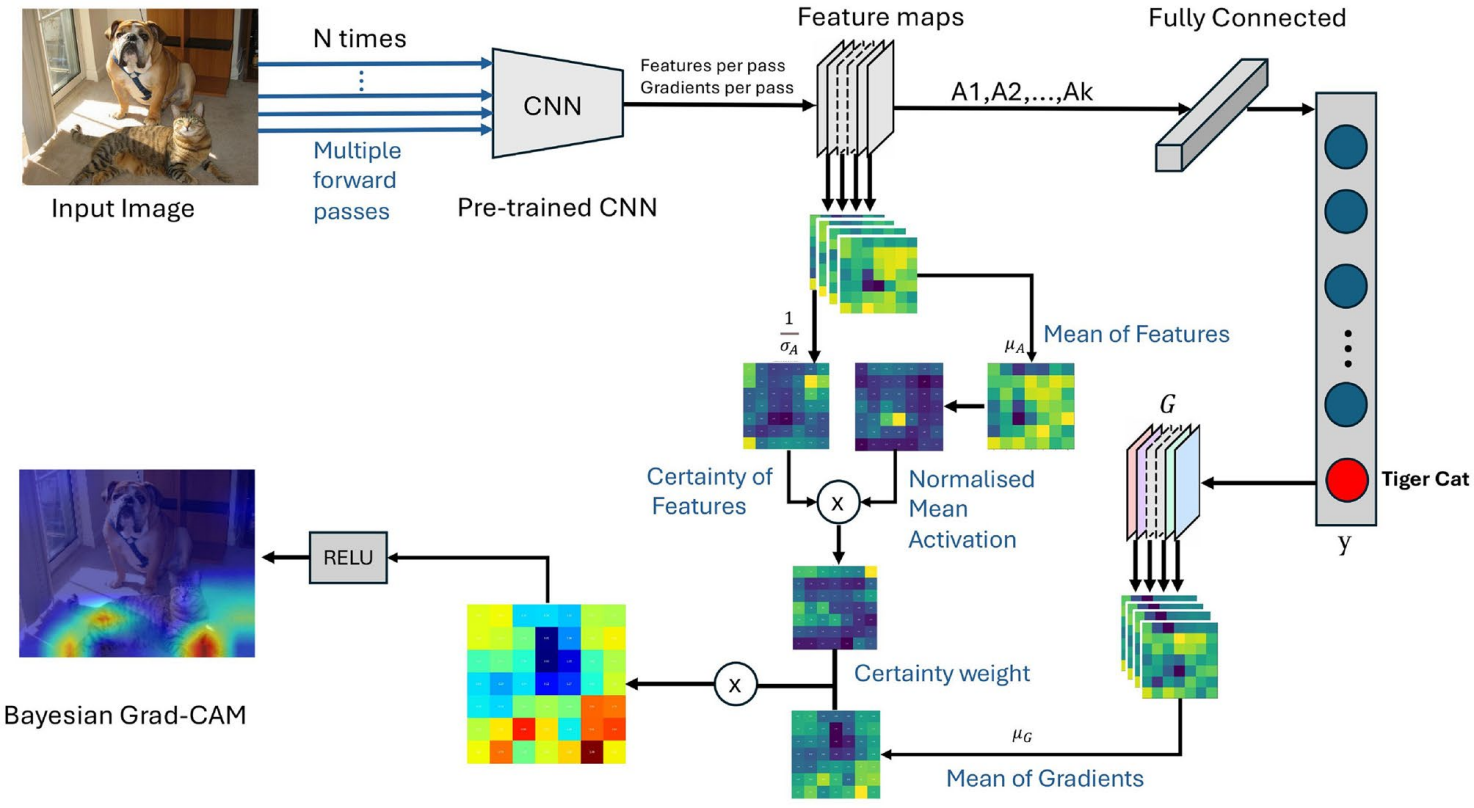
The Bayesian Grad-CAM overview depicts the process of generating a Class Activation Map (CAM) using a Bayesian approach. This method involves multiple forward passes to capture both feature maps and gradients, allowing for the calculation of their mean and standard deviation using Bayesian principles. The CAM is generated by multiplying the mean gradients with a certainty map. The contribution of each feature to the final CAM is determined by its certainty $C_A$.

The Bayesian Grad-CAM introduces an enhanced version of the traditional Grad-CAM by incorporating Bayesian pooling to improve robustness and reliability unlike traditional Grad-CAM, which uses global average pooling. The Bayesian Grad-CAM approach improves upon traditional Grad-CAM by integrating uncertainty into the visualisation of a model’s decision making process. During inference, the input is passed through the model multiple times, capturing feature maps at a designated target layer while the model operates in stochastic mode with dropout activated. Backpropagation is then employed to calculate the gradients of the output with respect to the feature maps at the target layer. These gradients reveal how minor changes in the feature maps affect the class score, emphasising the significance of each region.

This method gathers the feature maps and gradients from multiple forward passes using Bayesian statistics, computing both the mean and standard deviation. The process of averaging and modelling uncertainty leads to a more reliable Class Activation Map (CAM), as it takes into account variability in the model’s activations. Figure 2 illustrates how the Bayesian Grad-CAM method generates CAM. The mathematical formulation of this process is detailed in Equations (3)-(11).

3 $$\begin{aligned} \frac{\partial y}{\partial A_i} \end{aligned}$$

After multiple forward passes ($n$ passes), calculate the mean $\mu _A$ (4) and standard deviation $\sigma _A$ (5) of the feature maps across these passes, and the certainty of features ($C_A$) as calculated in Equation (6):

4 $$\begin{aligned} & \mu _A = \frac{1}{n} \sum _{i=1}^{n} A_i \end{aligned}$$

5 $$\begin{aligned} & \sigma _A = \sqrt{\frac{1}{n} \sum _{i=1}^{n} \left( A_i - \mu _A \right) ^2 + \epsilon } \end{aligned}$$

6 $$\begin{aligned} & C_A = \frac{1}{\sigma _A} \end{aligned}$$

where $\epsilon$ is a small constant to prevent division by zero.

Similarly, the mean of the gradients is computed as shown in Equation(7):

7 $$\begin{aligned} \mu _G = \frac{1}{n} \sum _{i=1}^n \frac{\partial y}{\partial A_i} \end{aligned}$$

The weights for the certainty map are computed by taking the activation value at each spatial location and normalising it across all feature maps by dividing by the sum of activations at that location, with a small epsilon added to ensure numerical stability, as shown in Equation (8):

8 $$\begin{aligned} w = \frac{\mu _A}{\sum _{c=1}^{C} \mu _A^{c} + \epsilon } \end{aligned}$$

Here, $\epsilon$ is a small constant to avoid division by zero. The weighted certainty map is calculated by multiplying the certainty of the features ($C_A$) by the normalised weights ($w$) and summing across the feature channels, as shown in Equation (9):

9 $$\begin{aligned} W_C = \sum _{c=1}^{C} \left( C_A^{c} \times w^{c} \right) \end{aligned}$$

The Class Activation Map (CAM) is obtained by multiplying the mean of gradients by the weighted certainty map, as shown in Equation (10):

10 $$\begin{aligned} CAM = \sum _{c=1}^{C} \mu _{G,c} \times W_{C,c} \end{aligned}$$

Finally, the ReLU function is applied to ensure non-negative values, as in Equation (11):

11 $$\begin{aligned} CAM_{\text {ReLU}} = \text {ReLU}(CAM) \end{aligned}$$

Algorithm 1 shows the full process. The core novelty in Bayesian pooling in Grad-CAM lies in aggregating feature maps and gradients across multiple forward passes, not just averaging them, but also considering their variability, as captured by the standard deviation. While traditional Grad-CAM assumes a deterministic model and calculates the contribution of each spatial location using the most likely (maximum a posteriori) gradients and activations, it does not account for uncertainty.

**Algorithm 1:**
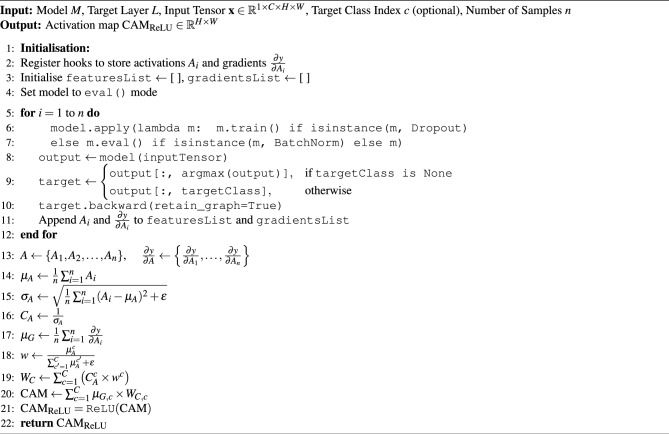
BayesianGradCAM: Sampling and Heatmap Generation

By using Bayesian approximation, this modified Grad-CAM accounts for uncertainty, leading to a more robust CAM. The result is a more trustworthy interpretation of which regions of the input image are important for the model’s prediction, as it considers both the average and the variability in feature maps across multiple forward passes. This method highlights the critical regions of the image most significant for class prediction, providing reliable and stable interpretations even when the model’s predictions lack high confidence. By effectively managing variability, the Bayesian Grad-CAM generates more informative visualisations.

**Fig. 3:**
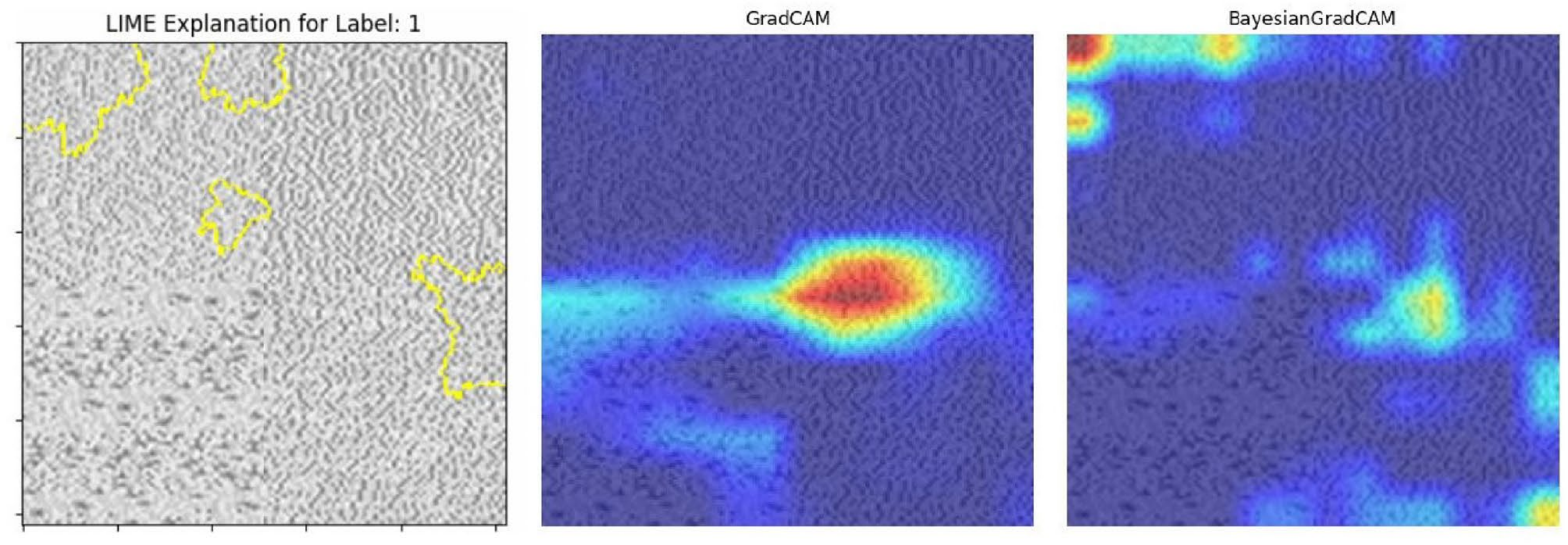
Bayesian Grad-CAM and Grad-CAM visualisations exhibit notable differences. By applying LIME to identify and highlight the regions most influential in the model’s prediction for a given class label, it is observed that Bayesian Grad-CAM primarily aligns with these key regions, indicating a closer correspondence to the features driving the model’s decision.

In Fig. 3, we compare the visualisations produced by traditional Grad-CAM, our proposed Bayesian Grad-CAM, and LIME. The results demonstrate that Bayesian Grad-CAM more consistently highlights the critical regions identified by LIME, indicating better localisation of the important features.

**Fig. 4:**
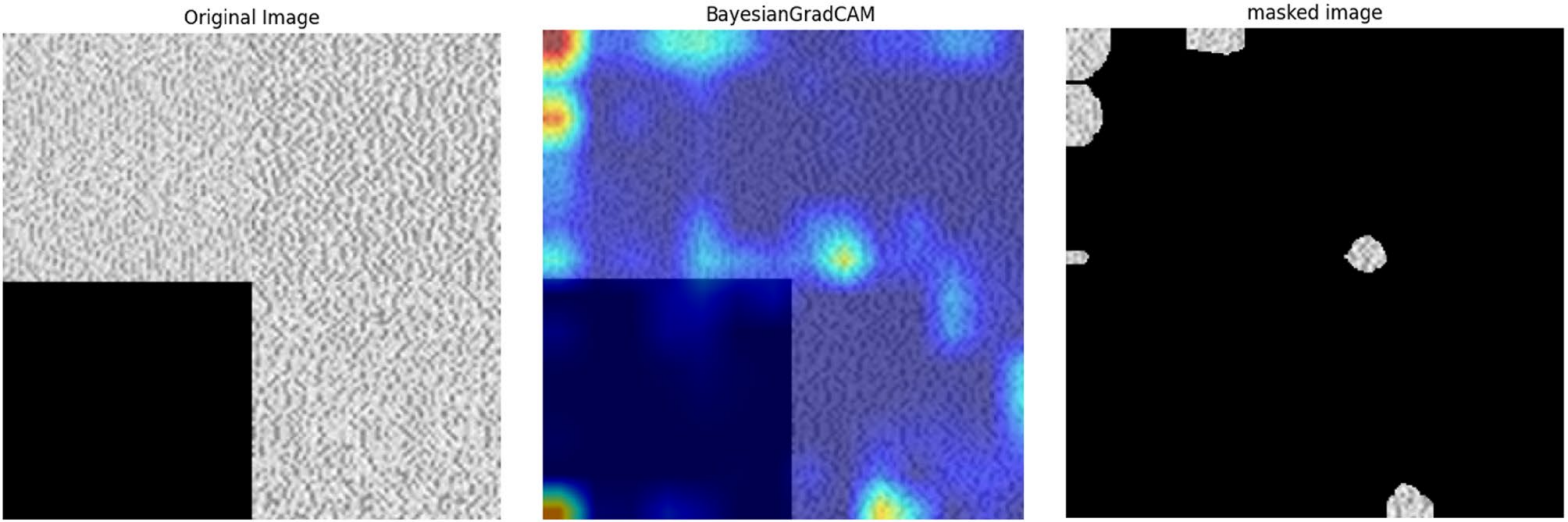
Process of key feature extraction by Bayesian Grad-Cam.

We first use Bayesian Grad-CAM on the target class to extract distinguishing regions from the images, resulting in highlighted areas that represent the key features of the class, as illustrated in Fig. 4. This process is repeated across all images for target classes, allowing us to capture their distinctive features. The extracted regions are saved for further analysis to better understand the distribution and significance of the key features identified by Bayesian Grad-CAM.

Next, we employ sliding windows to compare all the extracted regions of the same malware type with one another. We use a combination of Mean Squared Error (MSE), Histogram of Oriented Gradients (HOG) with Cosine Similarity, and Local Binary Patterns (LBP) to capture different aspects of the behaviour. MSE helps detect exact pixel-level differences, making it useful for finding small changes between nearly identical behaviours. HOG captures structural patterns, focusing on the overall flow or shape while being resilient to minor noise. LBP identifies textural differences, allowing us to detect local variations in behaviour. By combining these methods, we can accurately detect exact matches, structural similarities, and subtle behavioural variations.

MSE is first used to detect identical feature regions through a dynamic thresholding strategy. Rather than relying on a static similarity threshold, which can distort the similarity measurement, we use a dynamic threshold determined by the pixels being compared between features. As the extracted masks may include extensive black (background) areas, the threshold is scaled according to the non-black, information-rich portion of the image. The comparison is limited to non-black areas, allowing for consistent and accurate similarity evaluation regardless of the size or proportion of background pixels. This adaptive threshold adjusts to the image content, improving the accuracy of similarity measurements by concentrating on the relevant information and ignoring the background. This method reduces the influence of large uniform background areas. MSE is applied exclusively to non-black regions, focusing on the segmented content rather than the entire bounding box. This ensures that the similarity measure is sensitive to meaningful features and not influenced by background pixels, thereby enhancing its reliability across diverse conditions.

For MSE, we first calculate the total number of pixels in the template image. Next, we determine the percentage of black pixels in both images by counting the pixels with a value of zero and dividing this by the total pixel count. We proceed with the comparison only if both images contain at least 60 pixels and the percentage of black pixels does not exceed 50%.

To focus on informative parts of the image, we create masks to identify non-black pixels and combine them into an overlap mask, highlighting shared regions. We then extract and compare the non-background pixels from these areas. If MSE fails to find a match, HOG and LBP are then used to capture variations in the features. The dynamic threshold is computed as following Equation (12):

12 $$\begin{aligned} \text {Dynamic Threshold} = \text {threshold} \times \left( \frac{\text {Total Number of Non-Black Pixels in Overlapping Area}}{\text {Total Number of Pixels}} \right) \end{aligned}$$

We observed that the HOG value changes significantly depending on the feature size. When the compared features cover a smaller area, the computed HOG value is larger compared to those from larger features. This discrepancy leads to unstable comparisons, as larger areas may show more differences even when they share similar patterns. To address this issue, we define a dynamic threshold for HOG and also LBP similarity. Given HOG’s higher sensitivity to variations, it is assigned a lower base threshold compared to LBP. Equations (13) and (14) illustrate how we compute the threshold based on the size of features being compared:

13 $$\begin{aligned} & \text {HOG}_{\text {threshold}} = 0.4 + \frac{10}{\text {width} \times \text {height}} \end{aligned}$$

14 $$\begin{aligned} & \text {LBP}_{\text {threshold}} = 0.45 + \frac{10}{\text {width} \times \text {height}} \end{aligned}$$

We begin by identifying features for each malware type by analysing and comparing the extracted regions within images of the same type. When the compared regions differ in size, a sliding window is applied. With this, the smaller region is systematically moved across the larger one to enable localised comparison. If a high degree of similarity is identified, the matching area within the larger region is cropped using the corresponding coordinates and saved.

To maintain consistent spatial scale within a class, the smaller region can only be assigned the same label as the larger one if the differences in width and height are no greater than 4 pixels. We then limit our analysis to regions where the width is at least 30 pixels; any regions smaller than this threshold are excluded from grouping. This constraint ensures that the extracted feature patterns are structurally meaningful and facilitates the accurate retrieval of relevant API call names when converting pixel values back into string representations.

As previously stated, images are composed of four frames, with each frame representing a specific type of behavioural feature (network, file system, process, or registry API call names). Therefore, if the compared feature is not in the same frame as the first element of the class, it will not be added to the group.

Following this grouping process, we select the top 50 feature classes with the largest number of features. In the refinement stage, each feature is compared to the first element of each selected class and reassigned to the class it most closely resembles. This iterative reassignment improves clustering accuracy and reduces the likelihood of missing highly similar patterns during the initial grouping, resulting in a more robust and consistent classification of key malware features.

Among the top features selected for each class (after removing overlapping feature classes), we retain only the feature groups for each malware type that contain more than 500 objects. Since the feature classes are highly imbalanced, we redefine the threshold to balance the number of samples across classes. The resulting dataset, with the selected feature groups, includes 75 feature classes for 8 categories, as shown in Table 1. After converting the dataset to COCO^20^ format, we calculate the Intersection over Union (IoU) of bounding boxes within the same image to detect overlapping objects. For any overlapping objects, we retain the one that is most similar to the first object in its category. Using these results, we have created our own object detection dataset in COCO format for malware detection, focusing solely on essential features. Notably, no malware dataset currently exists for object detection.

**Fig. 5:**
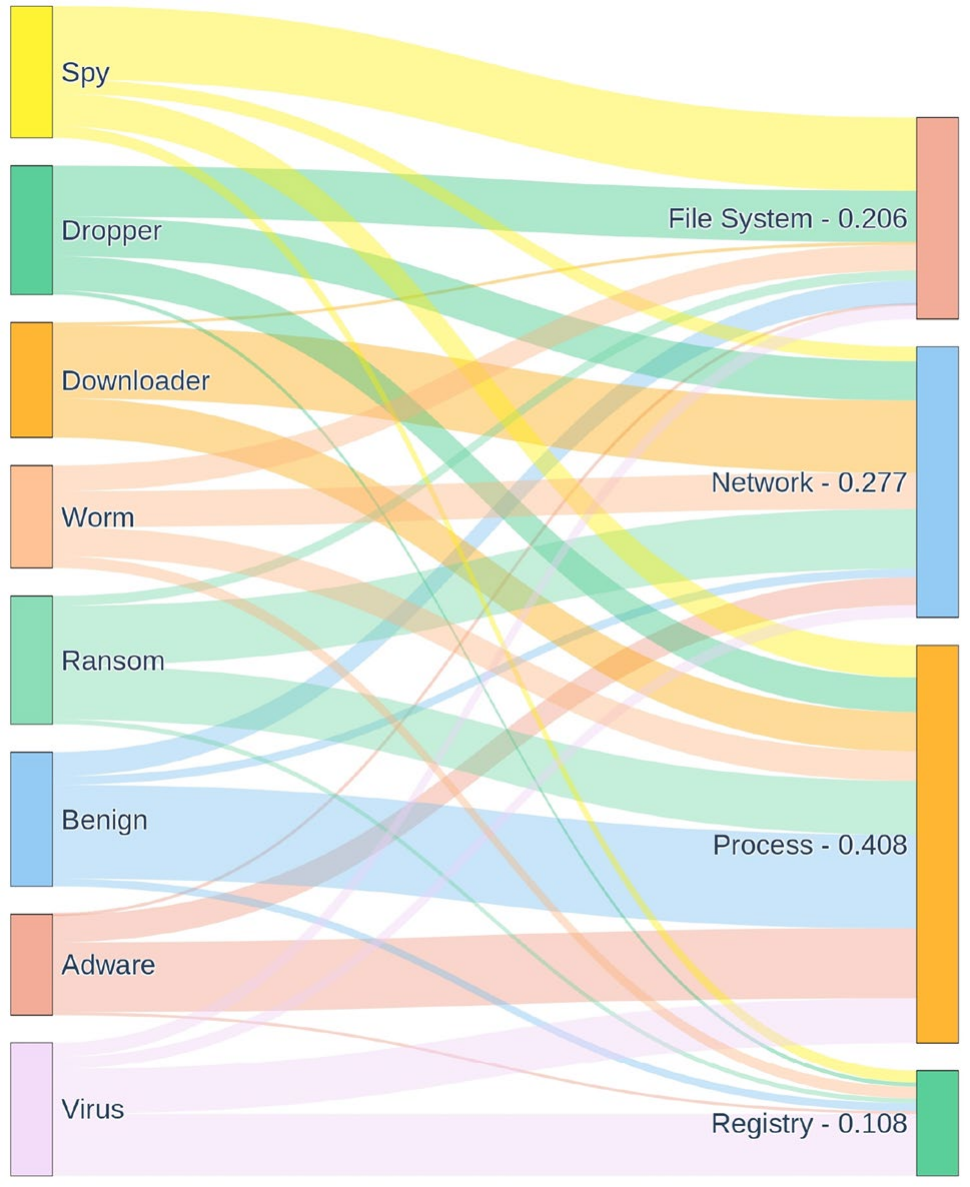
Sankey diagram of key feature type distribution across malware categories in the dataset. This diagram visualises the composition of feature groups for each malware supercategory, indicating the source of features based on API call types. Each malware category (e.g., virus, adware, ransomware) is linked to the four behavioural API call groups, Process, File System, Registry, and Network, from which the annotated object features were derived. The flow thickness reflects the proportion of feature annotations originating from each API group, highlighting how different malware families rely on distinct behavioural characteristics.

When analysing the key feature groups in our dataset, the Sankey diagram (Fig. 5) shows that each malware category includes feature groups derived from all four types of API calls, process, file system, registry, and network. However, process-related API features are especially prominent across categories, often emerging as the most influential in distinguishing behavioural patterns. In contrast, registry API calls appear less frequently as dominant key features. These insights highlight the varying discriminative power of API categories in characterising different malware behaviours.

### Feature localisation and detection

Using the COCO-based object detection dataset we developed, to identify key feature regions, we trained EfficientDet models (variants D1, D2, and D3) using different backbone architectures. Features localised and detected by the selected object detection model are utilised for quantitative malware composition analysis. To determine the proportion of malware types within a given sample, we applied a scoring strategy that accounts for both the diversity of detected features (i.e., distinct feature classes) and the weighted confidence scores associated with each detection.

Each malware type is defined by more than eight distinct features, making it essential to evaluate how many of these features are identified within a malware sample. Due to the repetitive nature of malware behaviour, the frequency or quantity of detected features reflects how often those features are predicted with the detection confidence, offering insights into the model’s focus and reliability. When quantifying features, it is also critical to account for confidence scores, as they indicate the model’s certainty in its predictions. To achieve this, predictions are weighted by their confidence scores and aggregated for each malware type, ensuring a more accurate representation of their significance.

**Table 1:** Number of annotations in the malware object detection dataset, grouped by malware supercategory. Each subtable presents the key features defined for that class. (B_F: Benign Feature, DW_F: Downloader Feature, A_F: Adware Feature, V_F: Virus Feature, S_F: Spyware Feature, W_F: Worm Feature, R_F: Ransomware Feature, D_F: Dropper Feature).

**Benign**		**Downloader**		**Adware**		**Virus**	
**Feature Class**	**No. Feature**	**Feature Class**	**No. Feature**	**Feature Class**	**No. Feature**	**Feature Class**	**No. Feature**
B_F1	627	DW_F1	568	A_F1	560	V_F1	615
B_F2	615	DW_F2	567	A_F2	552	V_F2	614
B_F3	600	DW_F3	563	A_F3	543	V_F3	599
B_F4	587	DW_F4	560	A_F4	537	V_F4	597
B_F5	577	DW_F5	546	A_F5	535	V_F5	595
B_F6	571	DW_F6	519	A_F6	526	V_F6	575
B_F7	566	DW_F7	516	A_F7	513	V_F7	562
B_F8	556	DW_F8	497	A_F8	503	V_F8	554
B_F9	556	DW_F9	494			V_F9	540
B_F10	547					V_F10	518

**Spyware**		**Worm**		**Ransomware**		**Dropper**	
**Feature Class**	**No. Feature**	**Feature Class**	**No. Feature**	**Feature Class**	**No. Feature**	**Feature Class**	**No. Feature**
S_F1	603	W_F1	622	R_F1	581	D_F1	603
S_F2	586	W_F2	585	R_F2	581	D_F2	600
S_F3	586	W_F3	566	R_F3	570	D_F3	598
S_F4	572	W_F4	545	R_F4	565	D_F4	592
S_F5	570	W_F5	542	R_F5	562	D_F5	588
S_F6	569	W_F6	521	R_F6	549	D_F6	581
S_F7	566	W_F7	520	R_F7	538	D_F7	576
S_F8	546	W_F8	461	R_F8	534	D_F8	558
S_F9	535			R_F9	526	D_F9	548
S_F10	534			R_F10	503	D_F10	535

While the quantity of confidence predicted features provides insight into the reliability characteristics of malware, diversity offers a deeper understanding of the range of actions and intentions associated with different malware types. To balance these aspects, we combine both the weighted confidence scores of predictions, which prioritise high confidence scores to ensure that more reliable predictions are given greater weight, and the normalised number of distinct features (i.e., diversity). This calculation accounts for both the confidence of the predictions and the diversity of features present in each sample for a given category.

As outlined in Equation (15), the score for each identified category is calculated by applying weights to prioritise higher-confidence scores. Equation (16) provides likelihood of each detected class combining weighted confidence score with the normalised diversity, which is based on the maximum number of distinct feature classes among the detected categories. Here, $\text {diversity}_{\text {category}}$ is the count of distinct features detected for a malware type, representing diversity, while $\sum _{i=1}^{N} f(\text {conf}_i)$ is the aggregated and weighted confidence score for all predictions associated with a malware type. This scoring mechanism helps prioritise categories, enabling the identification of the most critical malware types or features for further investigation, thereby optimising detection and analysis efforts.

This dual-weighted approach effectively integrates both diversity and quantity into the decision-making process, enabling a more comprehensive and reliable analysis of malware behaviour.

15 $$\begin{aligned} & f(\text {conf}_i) = {\left\{ \begin{array}{ll} 0.6 \cdot \text {conf}_i, & \text {if } \text {conf}_i> 0.5 \\ 0.5 \cdot \text {conf}_i, & \text {if } \text {conf}_i> 0.4 \\ 0.4 \cdot \text {conf}_i, & \text {if } \text {conf}_i> 0.3 \\ 0.3 \cdot \text {conf}_i, & \text {if } \text {conf}_i> 0.2 \\ 0.1 \cdot \text {conf}_i, & \text {otherwise} \end{array}\right. } \end{aligned}$$

16 $$\begin{aligned} & \text {Score}_{\text {category}} = \sum _{i=1}^{N} f(\text {conf}_i) \times \frac{\text {diversity}_{\text {category}}}{\text {max}\_\text {diversity}} \end{aligned}$$

We use Bayes’ Theorem for scoring labels, enabling a balanced and context-aware classification by integrating prediction scores with class distributions. This approach prioritises high-confidence predictions while reducing the impact of uncertain detections.

Specifically, Bayes’ Theorem is employed for both binary and multi-label classification. The posterior probability, for a given category is calculated by multiplying the likelihood of its detection, Equation (17), by its prior probability $P(\text {category}_i)$, defined by the frequency of that category in the dataset. This product is then normalised by the total probability across all detections, as described in Equation (18).

17 $$\begin{aligned} P(\text {detections} \mid \text {category}_i) \approx \text {Score}_{\text {category}_i} \end{aligned}$$

The final classification is based on the highest posterior probability. If multi-label classification predicts benign, but binary classification predicts malware, we select the second-highest posterior probability to refine the classification decision.

18 $$\begin{aligned} P(\text {category}_i \mid \text {detections}) = \frac{P(\text {detections} \mid \text {category}_i) \cdot P(\text {category}_i)}{\sum \limits _{j=1}^{N} P(\text {detections} \mid \text {category}_j) \cdot P(\text {category}_j)} \end{aligned}$$

In imbalanced datasets, Bayes’ Theorem adjusts for class importance using prior probabilities, ensuring that rare or underrepresented categories are properly accounted for, thereby preventing overfitting to dominant classes. Moreover, its interpretability allows us to trace and justify classification decisions, making it particularly valuable in malware classification.

### Understanding detection via multi-agent-based reverse engineering

As part of our goal to develop a visually explainable malware detection system, we extract and report the corresponding API call names associated with the detected feature regions for each analysed image, serving as the final step in our detection process. To make the predictions understandable to end users, we display each detected feature region alongside its corresponding class label. This enhances explainability by illustrating which features influence the prediction of a specific class.

Analysing these decoded features alongside their predicted labels provides valuable insight into the behavioural intent of the malware. To support analysts in interpreting these results, we employ a large language model (LLM) to generate clear and comprehensible explanations. However, LLMs present a significant challenge known as hallucination, in which the model produces responses that appear coherent and plausible, yet are factually incorrect or entirely fabricated. This issue stems from the way LLMs are trained, by learning from vast amounts of data and identifying statistical patterns, without any grounded understanding of the real world. As a result, LLMs are unable to reliably distinguish truth from fiction^21^.

To enhance the reasoning capabilities of LLMs, Wei et al.^22^ introduced chain-of-thought (CoT) prompting, which guides models through a coherent sequence of intermediate reasoning steps in a few-shot format. Their findings show that CoT prompting enables sufficiently large models to perform complex reasoning tasks more effectively than smaller models or traditional prompting methods. It also improves interpretability and performance while requiring only a few in-context examples.

To address the limitations of conventional CoT, particularly the lack of iterative refinement and external validation, Sanwal^23^ proposed Layered Chain-of-Thought (Layered-CoT) Prompting in multi-agent LLM systems. This approach decomposes reasoning into distinct stages, each producing a partial output that is independently verified using external resources such as domain-specific databases, knowledge graphs, or expert feedback via interactive dialogue. Unlike standard CoT, which validates only the final output, Layered-CoT applies verification throughout the reasoning pipeline. This layered validation enhances factual accuracy, coherence, and transparency. When combined with a multi-agent architecture, it gains modularity, enabling specialised agents to perform tasks such as retrieval, verification, and user interaction, thereby forming a more robust and adaptable framework. Multi-agent LLM systems further support collaborative reasoning and cross-verification among agents, enabling more dynamic and context-aware problem-solving.

According to Ghosmar and Dhal^24^, utilising multiple specialised agents within a coordinated pipeline can significantly reduce hallucinations in Large Language Models (LLMs). By following the initial content generation with structured review stages, designed to insert disclaimers, refine speculative language, and minimise potentially misleading factual claims, the approach leads to a clear reduction in hallucination rates. In multi-agent collaboration for LLM cross-examination, each agent is responsible for a specific task. These tasks may include aggregation, scoring, judgement, revision, and other roles that collectively enhance the reliability and factual consistency of the model’s output.

Among the available LLM models, LLaMA and Mistral are freely distributed for research and commercial use. LLaMA 3 incorporates 8 billion parameters along with improved attention mechanisms and a broader context window, enabling it to manage sophisticated reasoning challenges that involve detailed, long-term contextual understanding^25^. The strength of Mistral-7B-Instruct-v0.3 lies in its capacity to generate summaries that are both varied and highly readable. It produces outputs that are not only precise but also clear and easy for readers to comprehend^26^.

Building on these developments, we propose a multi-agent module extending the Mistral-7B-Instruct-v0.3 model to improve the reliability and factual consistency of malware behaviour explanations. Each agent is guided by a role-specific prompt, employing in-context learning and zero-shot instruction, which removes the necessity for task-specific fine-tuning. Prior work by Reynolds and McDonell^27^ showed that well-crafted zero-shot prompts may outperform few-shot prompts, as the inclusion of examples can lead models to interpret them narratively rather than as categorical instructions^28^. To reduce hallucinations and ground the reasoning process, we generate a reference mapping of the MITRE ATT&CK framework^29^. These grounded inputs support domain-aware explanation refinement at intermediate stages.

**Fig. 6:**
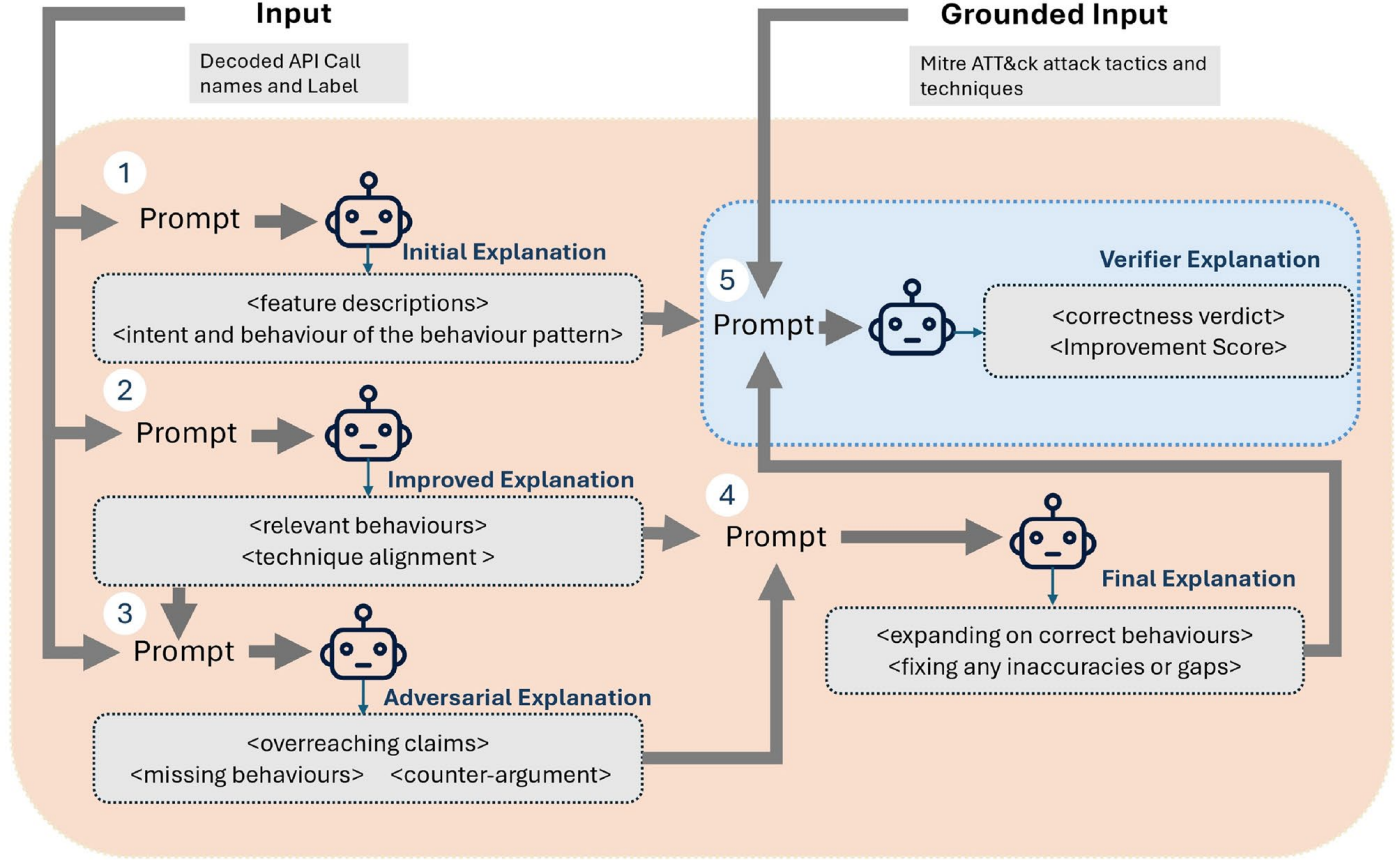
Multi-agent pipeline for malware behaviour analysis. The Initial Generator produces a baseline explanation from the observed API features, serving solely as a verification benchmark. The Reviewer Agent independently develops an explanation grounded in the observed API features and aligns behaviours with official MITRE ATT&CK technique names. The Adversarial Agent critically evaluates this explanation, identifying omissions or overclaims. The Consensus Agent integrates these perspectives to produce a coherent final explanation. Finally, the Verifier Agent assesses the output against the baseline, providing a Correctness Verdict and an Improvement Score.

The first stage of the framework (illustrated in Fig. 6) generates improved explanations through a structured multi-agent process. It begins with an initial baseline explanation derived from the observed API features, retained solely as a reference point for verification. Independently, the Reviewer Agent develops an explanation grounded in the observed API features, interpreting their functional implications and aligning the identified behaviours with official MITRE ATT&CK technique names. This explanation is then critically evaluated by the Adversarial Agent, whose role is to identify overgeneralised statements, unsupported assumptions, or omissions. A Consensus Agent then synthesises the outputs from both the Reviewer and Adversarial agents, producing a final explanation that is more accurate, coherent, and balanced.

Each agent in the pipeline has a specialised function. The Reviewer incorporates domain-specific knowledge to clarify the behavioural intent of the observed API features. the Adversarial Agent critically challenges the Reviewer’s output by identifying overgeneralised claims, omissions, or unsupported assumptions. This adversarial framework helps mitigate individual model biases and facilitates a more nuanced, context-aware evaluation of LLM performance^30^. The Consensus Agent integrates both viewpoints into a unified explanation that reflects precision and completeness.

To assess the quality of the final output, a Verifier Agent is employed, powered by the Meta-LLaMA-3-8B-Instruct model. Unlike the other agents, the Verifier has access to grounded reference materials, specifically the MITRE ATT&CK framework^29^. It uses this information to evaluate whether the explanation accurately captures system-level behaviours, aligns with documented adversarial techniques, and constitutes a meaningful improvement relative to an initial baseline explanation. The Verifier provides a structured assessment including a Correctness Verdict, an Improvement Score, and a Justification, thereby offering transparent and evidence-based validation. The consensus explanation incorporates adversarial feedback by critically reassessing the Reviewer’s assumptions and adopting a more cautious, evidence-based, and context-aware interpretation of observed behaviours. For example, while the Reviewer links file creation and registry access to persistence or reconnaissance, the Adversarial Agent points out that such actions are also common in legitimate software, such as installers or configuration utilities. In response, the consensus explanation acknowledges the dual-use nature of these APIs and treats them as potential indicators of malicious activity only when corroborated by additional, stronger signals. This more nuanced interpretation avoids overgeneralisation and promotes a balanced, evidence-driven assessment of the sample’s intent.

The Verifier supports this balanced and accurate assessment, stating that the explanation thoroughly covers system-level behaviours, aligns with relevant MITRE ATT&CK techniques, and directly addresses the Adversarial Agent’s concerns. Overall, it is recognised as a strong refinement, as reflected in the assigned improvement score of 8.

## Results

In this section, we present experimental results evaluating the effectiveness of the proposed DECODE framework. We first report a series of ablation studies that analyse the contribution of individual components. We then provide the final comparative evaluation against CNN baselines in both binary and multi-label settings, followed by qualitative analyses to demonstrate interpretability.

### Ablation study

#### Dataset cleaning

To evaluate the effectiveness of repeated-group cleaning on dataset quality and downstream model performance, we conducted an ablation study using different hyperparameters settings for the sliding-window process. Three configurations were compared:

1. **SWSD-MSL10-RGC**: Minimum Segment Length = 10, repeated-group cleaning enabled,
2. **SWSD-MSL5-RGC**: Minimum Segment Length = 5, repeated-group cleaning enabled, and
3. **SWSD-NoRGC**: Repeated-group cleaning disabled (baseline).

All experiments employed identical training and validation splits to ensure direct comparability. The dataset comprised the following per-class sample counts (training, validation): adware (426, 107), ransomware (675, 169), spyware (669, 168), benign (762, 191), worm (625, 157), downloader (712, 178), virus (600, 151), and dropper (517, 130).

Among the tested configurations, the model trained with repeated-group cleaning and a minimum segment length of 10 (SWSD-MSL10-RGC) consistently achieved the highest values across all evaluation metrics. The complete set of comparative metrics and extended analyses are available in the project repository. These findings demonstrate that applying repeated-group cleaning with a larger segment size improves dataset representativeness by reducing redundancy while preserving meaningful behavioural semantics.

#### Feature learning

We first compared candidate model architectures (VGG16, ResNet34/50/101, and InceptionV4) to determine the most effective backbone for feature extraction (Table 2). The dataset is partitioned into two subsets, a training set comprising 5,076 samples and a validation set containing 1,273 samples. The training set is distributed across eight categories as follows: Adware (438), Virus (676), Ransomware (675), Worm (627), Spyware (669), Dropper (517), Downloader (712), and Benign (762). The validation set follows a similar distribution, consisting of Adware (110), Virus (170), Ransomware (169), Worm (157), Spyware (168), Dropper (130), Downloader (178), and Benign (191). VGG16 achieved superior performance across accuracy, F1 score, MCC, and ROC AUC, and was therefore selected as the primary model for subsequent experiments.

To determine the optimal dropout rate, models with dropout values of 0.3 and 0.5 were evaluated. The 0.3 configuration consistently outperformed 0.5 across all metrics (Table 2) and was adopted in the final model. Although the dropout-enhanced VGG16 showed a slight reduction in accuracy compared with the non-dropout baseline, it achieved a substantially lower false positive rate (FPR).

**Table 2:** NPV : Negative Predictive Value; FPR : False Positive Rate; FNR : False Negative Rate; MCC : Matthews Correlation Coefficient. All models were trained and evaluated on the generated malware image dataset. Metric values are reported as proportions between 0 and 1. Higher values for Accuracy, Precision, Recall, F1 Score, ROC AUC, Specificity, NPV, Balanced Accuracy, and MCC indicate better performance, while lower values for FPR and FNR are preferred. All models used the softmax loss, except for the first two VGG16 models, which were trained with a Joint Loss approach. For both VGG16 with Joint Loss and VGG16 with softmax loss, dropout layers were applied as indicated. All reported metrics fell within expected ranges, with no NaN or missing entries observed. Distributions of Precision, Recall, and MCC across samples showed consistent trends without outliers, indicating no statistical anomalies or data integrity issues during model evaluation.

**Model**	**Accuracy**	**Precision**	**Recall**	**F1 Score**	**ROC AUC**	**Specificity**	**NPV**	**FPR**	**FNR**	**Balanced Accuracy**	**MCC**
VGG16 with Joint Loss	0.8264	0.8287	0.8263	0.8253	0.9007	0.9001	0.8832	0.0999	0.1737	0.8632	0.7928
VGG16 with Joint Loss + Dropout (0.3)	0.8052	0.8218	0.8182	0.8194	0.8959	0.9720	0.9721	0.0280	0.1922	0.8899	0.7770
VGG16 with Softmax Loss + Dropout (0.3)	0.8036	0.8093	0.8028	0.8054	0.8873	0.8093	0.8028	0.1907	0.1972	0.8060	0.7748
VGG16 with Joint Loss + Dropout (0.5)	0.7894	0.7969	0.7899	0.7928	0.8798	0.9445	0.9596	0.0554	0.2251	0.8597	0.6949
ResNet34 with CBAM and Softmax Loss	0.7683	0.7906	0.7662	0.7715	0.8664	0.7906	0.7662	0.2094	0.2338	0.7784	0.7357
ResNet50 with SENet and Softmax Loss	0.7510	0.7908	0.7448	0.7566	0.8543	0.7908	0.7448	0.2092	0.2552	0.7678	0.7173
InceptionV4 with Softmax Loss	0.7416	0.7547	0.7435	0.7438	0.8531	0.7547	0.7435	0.2453	0.2565	0.7491	0.7050
ResNet101 with Softmax Loss	0.7211	0.7389	0.7202	0.7190	0.8400	0.7389	0.7202	0.2611	0.2798	0.7296	0.6830
ResNet34 with SENet and Softmax Loss	0.7101	0.7653	0.7084	0.7196	0.8333	0.7653	0.7084	0.2347	0.2916	0.7368	0.6750
ResNet50 with Softmax Loss	0.6544	0.6799	0.6578	0.6528	0.8040	0.6799	0.6578	0.3201	0.3422	0.6688	0.6067
ResNet34 with Softmax Loss	0.6630	0.7025	0.6548	0.6587	0.8030	0.7025	0.6548	0.2975	0.3452	0.6786	0.6179
ResNet50 with CBAM and Softmax Loss	0.6512	0.6859	0.6511	0.6510	0.8004	0.6859	0.6511	0.3141	0.3489	0.6685	0.6045
ResNet101 with CBAM and Softmax Loss	0.6222	0.6761	0.6251	0.6253	0.7853	0.6761	0.6251	0.3239	0.3749	0.6506	0.5739
ResNet101 with SENet and Softmax Loss	0.6159	0.6576	0.6145	0.6223	0.7795	0.6576	0.6145	0.3424	0.3855	0.6361	0.5613

**Fig. 7:**
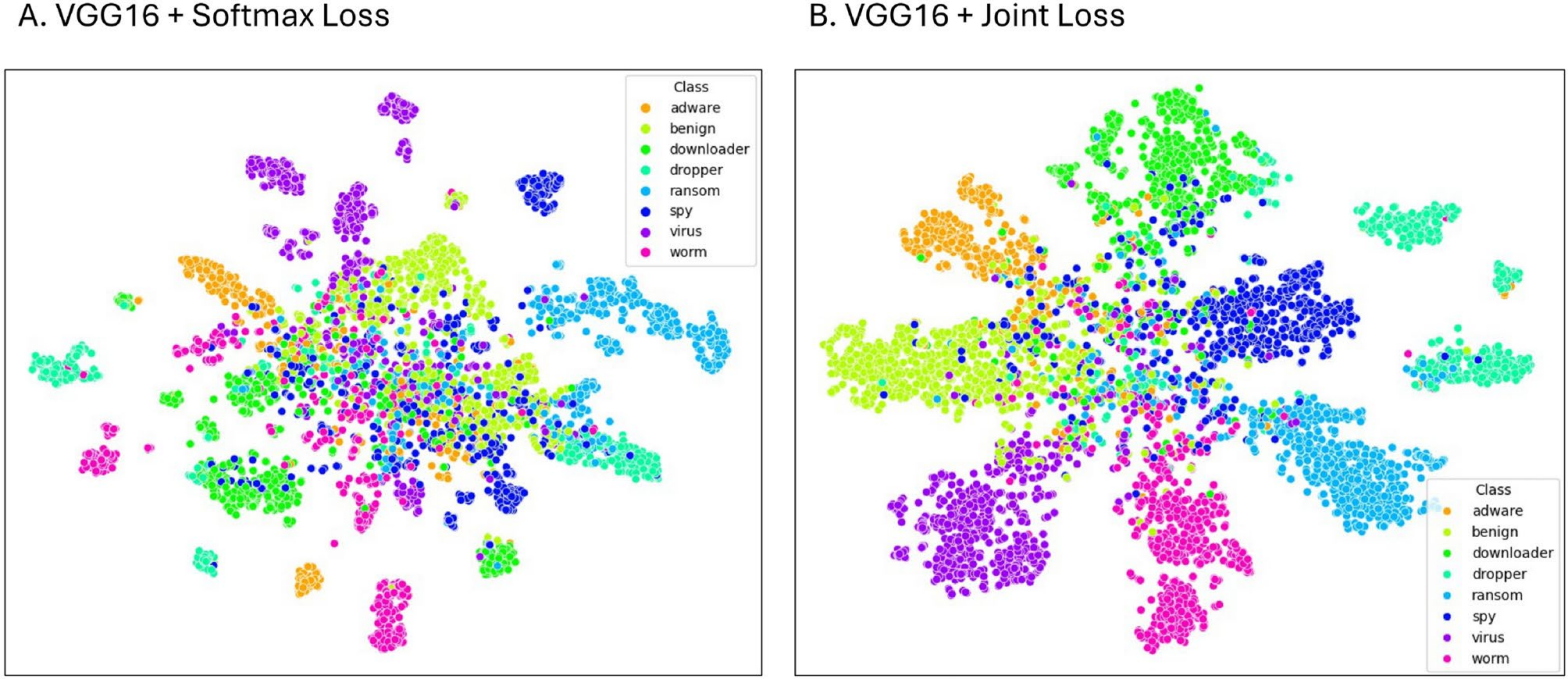
t-SNE plots illustrating the image feature representations extracted by two VGG16 models trained on the malware dataset. Plot A corresponds to training with softmax loss, while Plot B corresponds to training with joint loss. The joint loss model shows enhanced inter-class separation and reduced intra-class variance.

We then investigated the impact of loss function design. A joint loss was compared against softmax alone. The joint loss produced superior performance by improving inter-class separability and reducing intra-class variance. This effect is evident in the t-SNE visualisations (Fig. 7), where the joint loss model (Plot B) exhibits clearer separation of classes compared with the softmax-only baseline (Plot A). Well-defined clusters are observed for Dropper, Ransomware, and Virus, while Spyware and Benign remain poorly separated, reflecting their behavioural overlap with other classes.

#### Grad-CAM vs Bayesian Grad-CAM

**Fig. 8:**
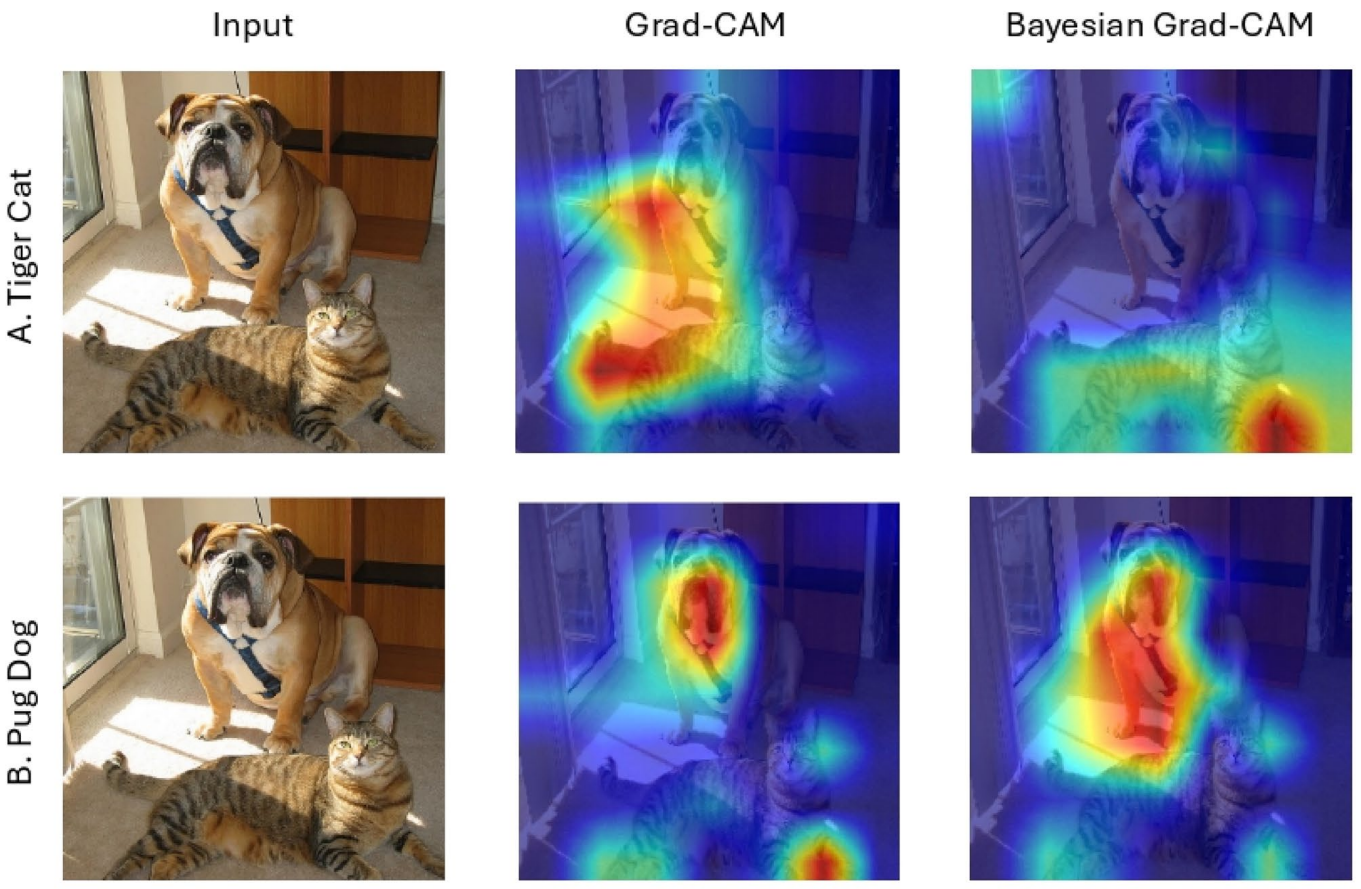
Comparison of Grad-CAM and Bayesian Grad-CAM visualisations on the ImageNet dataset. Visualisations are generated using a customised ResNet-50 architecture, in which a Dropout layer (with probability 0.5) is inserted after the second BatchNorm layer (bn2) in each Bottleneck block. Panel A corresponds to the target class tiger cat: Grad-CAM highlights both dog and cat regions, indicating class ambiguity, while Bayesian Grad-CAM more precisely focuses on cat-specific features, demonstrating improved class specificity. Panel B corresponds to the target class pug dog: Bayesian Grad-CAM broadens the highlighted area across the full dog region and suppresses attention to background and irrelevant objects.

To assess the impact of uncertainty modelling on visual explanation quality, we compared traditional Grad-CAM with the proposed Bayesian Grad-CAM. Figure 8 provides a comparative analysis of traditional Grad-CAM and our newly developed Bayesian Grad-CAM techniques on the ImageNet dataset. The visualisations reveal that while traditional Grad-CAM highlights large areas encompassing both key object features and background elements, Bayesian Grad-CAM offers more targeted activation. Specifically, Bayesian Grad-CAM zeroes in on the essential features of objects, significantly diminishing attention to the background and non-relevant regions. This improved focusing ability of Bayesian Grad-CAM highlights its superiority in delivering clearer and more precise interpretations of model predictions.

**Fig. 9:**
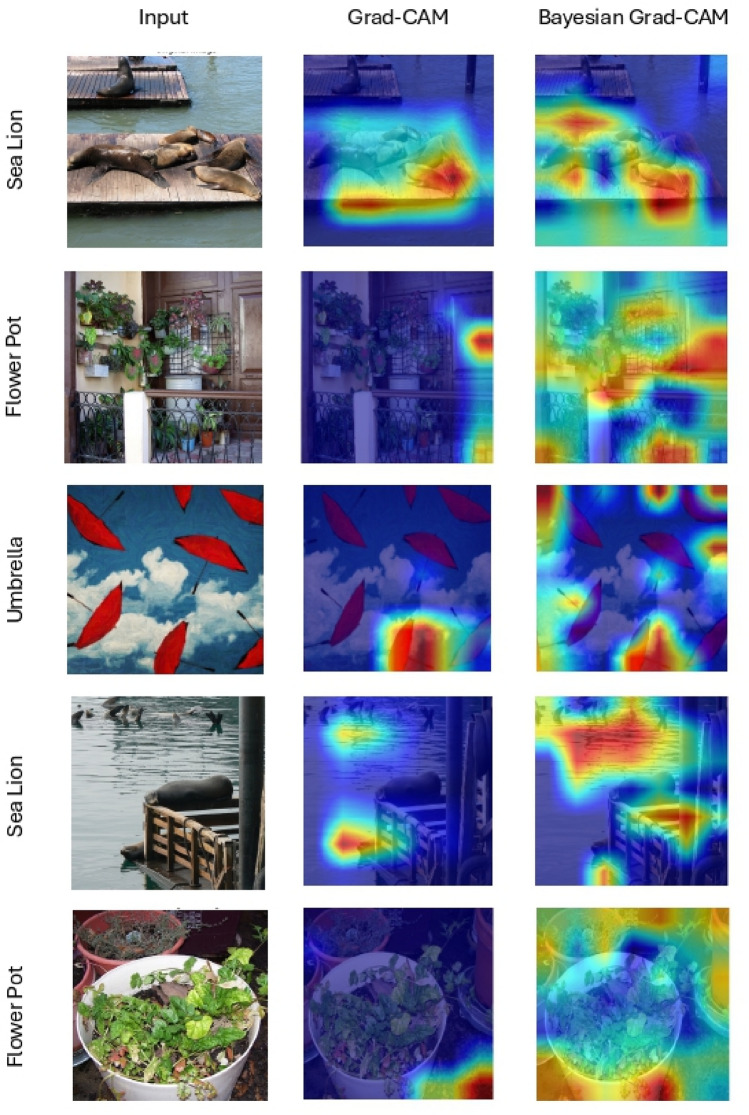
Comparison of Grad-CAM and Bayesian Grad-CAM visualisations using the custom ResNet-50 on ImageNet validation data. Bayesian Grad-CAM provides clearer, more stable attribution maps, accurately highlighting multiple instances of the target class, outperforming standard Grad-CAM in both precision and consistency.

A further comparison on multi-object images (Fig. 9) shows that Grad-CAM often fails to accurately localise all relevant objects. In contrast, Bayesian Grad-CAM offers a more stable and comprehensive focus, effectively identifying and highlighting nearly all instances of the target class. This capability is especially critical in applications where the exclusion of relevant objects could undermine the reliability and completeness of the model’s explanation. These qualitative results demonstrate that incorporating uncertainty into the pooling process improves both spatial precision and class specificity of attribution maps.

To qualitatively assess the capability of Bayesian Grad-CAM to emphasise class-specific features, we created two datasets by segmenting images using heatmaps from Grad-CAM and Bayesian Grad-CAM, respectively. A custom VGG16 model was trained on each dataset under identical training and validation distributions. The model trained on Bayesian Grad-CAM-segmented images consistently outperformed the one trained on Grad-CAM-segmented images. This finding suggests that Bayesian Grad-CAM more effectively captures discriminative category features, and that removing irrelevant information from the training data can enhance classification performance. Full comparative results are provided in the associated GitHub repository.

### Comparative evaluation with baseline classifiers

We evaluated the EfficientDet object detection model on our custom malware dataset. Among the tested variants, EfficientDet-D2, which employs the EfficientNetV2-S backbone, demonstrated the better overall accuracy. A detailed comparative analysis is provided in the accompanying GitHub repository. To evaluate the effectiveness of the proposed DECODE framework, which integrates object detection with quantitative multi-label behavioural analysis, we conducted comparative experiments against several baseline CNN classifiers. These CNN models were fine-tuned on the same domain-specific malware behaviour image dataset commonly used in malware image classification tasks, thereby ensuring their relevance to the problem domain.

The evaluation dataset comprised a total of 5,708 training samples and 1,177 validation samples, distributed across eight categories. The training set included 635 samples of spyware, 634 ransomware, 385 adware, 730 benign, 646 virus, 573 worm, 588 downloader, and 517 dropper. The validation set contained 161 spyware, 156 ransomware, 96 adware, 185 benign, 162 virus, 143 worm, 146 downloader, and 128 dropper samples.

Across both binary and multi-label classification tasks, DECODE consistently outperformed the baseline models. By assigning class labels based on the highest proportion of behavioural traits detected within each image, the framework achieved competitive classification performance while maintaining interpretability. These results highlight the advantages of incorporating behaviour-aware visual features into the decision-making process for malware analysis.

**Table 3:** Binary Classification Performance Analysis Between DECODE and Baseline Methods. The metrics include ACC (Accuracy), FPR (False Positive Rate), FNR (False Negative Rate), and MCC (Matthews Correlation Coefficient). All values range from 0 to 1.

**Model**	**ACC**	**F1 Score**	**FNR**	**FPR**	**MCC**
DECODE	0.9380	0.9619	0.0655	0.0432	0.8032
InceptionResNetV2	0.9200	0.9599	0.0343	0.2486	0.7370
ResNet18	0.9170	0.9562	0.0301	0.1910	0.7023
InceptionV4	0.9170	0.9441	0.0484	0.1892	0.7414
ResNet34	0.9140	0.9568	0.0479	0.2171	0.7181
VGG16	0.9060	0.9520	0.0585	0.1946	0.7137
ResNet50	0.8880	0.9455	0.0872	0.1711	0.5951

In the context of multi-label classification, we use evaluation metrics such as Hamming Loss, F1-Score, and overall accuracy (ACC). In addition, we evaluate label-based accuracy to assess performance across individual classes. Hamming Loss, which measures the ratio of misclassified labels to the total number of labels, is particularly useful in the multi-label setting. For binary classification, we use Accuracy (ACC), F1 Score, False Positive Rate (FPR), and Matthews Correlation Coefficient (MCC).

**Table 4:** Multi-Label Classification Performance Comparison Between DECODE and Baseline Methods. ACC: Accuracy; HL: Hamming Loss; Class-Based Accuracy is reported separately for Adware, Benign, Downloader, Dropper, Ransomware, Spyware, Virus, and Worm. All metrics range from 0 to 1.

**Metric/Label**	**DECODE**	**InceptionV4**	**ResNet18**	**ResNet34**	**VGG16**	**ResNet50**	**IncepResNetV2**
ACC	0.8513	0.8181	0.8173	0.8088	0.8011	0.7162	0.8113
F1-Score	0.8515	0.8202	0.8202	0.8121	0.8036	0.7155	0.8144
HL	0.0372	0.1818	0.1827	0.1912	0.1988	0.2838	0.1886
Adware	0.9785	0.9636	0.9687	0.9632	0.9696	0.9227	0.9667
Benign	0.9382	0.9286	0.9206	0.9078	0.9165	0.9069	0.9300
Downloader	0.9554	0.9491	0.9562	0.9374	0.9514	0.9387	0.9462
Dropper	0.9825	0.9800	0.9742	0.9731	0.9736	0.9142	0.9680
Ransomware	0.9683	0.9647	0.9588	0.9576	0.9560	0.9580	0.9627
Spyware	0.9310	0.9185	0.9169	0.8855	0.9213	0.8942	0.9235
Virus	0.9769	0.9665	0.9604	0.9460	0.9468	0.9445	0.9612
Worm	0.9747	0.9743	0.9635	0.9746	0.9736	0.9567	0.9772

For binary classification accuracy, as shown in Table 3, DECODE improves performance by approximately 1.8%, increasing from 92% with InceptionResNetV2, the highest-performing baseline model, to 95.8%. Regarding the Matthews Correlation Coefficient (MCC), DECODE achieves the highest score, providing a balanced and robust evaluation of binary classification performance.

**Fig. 10:**
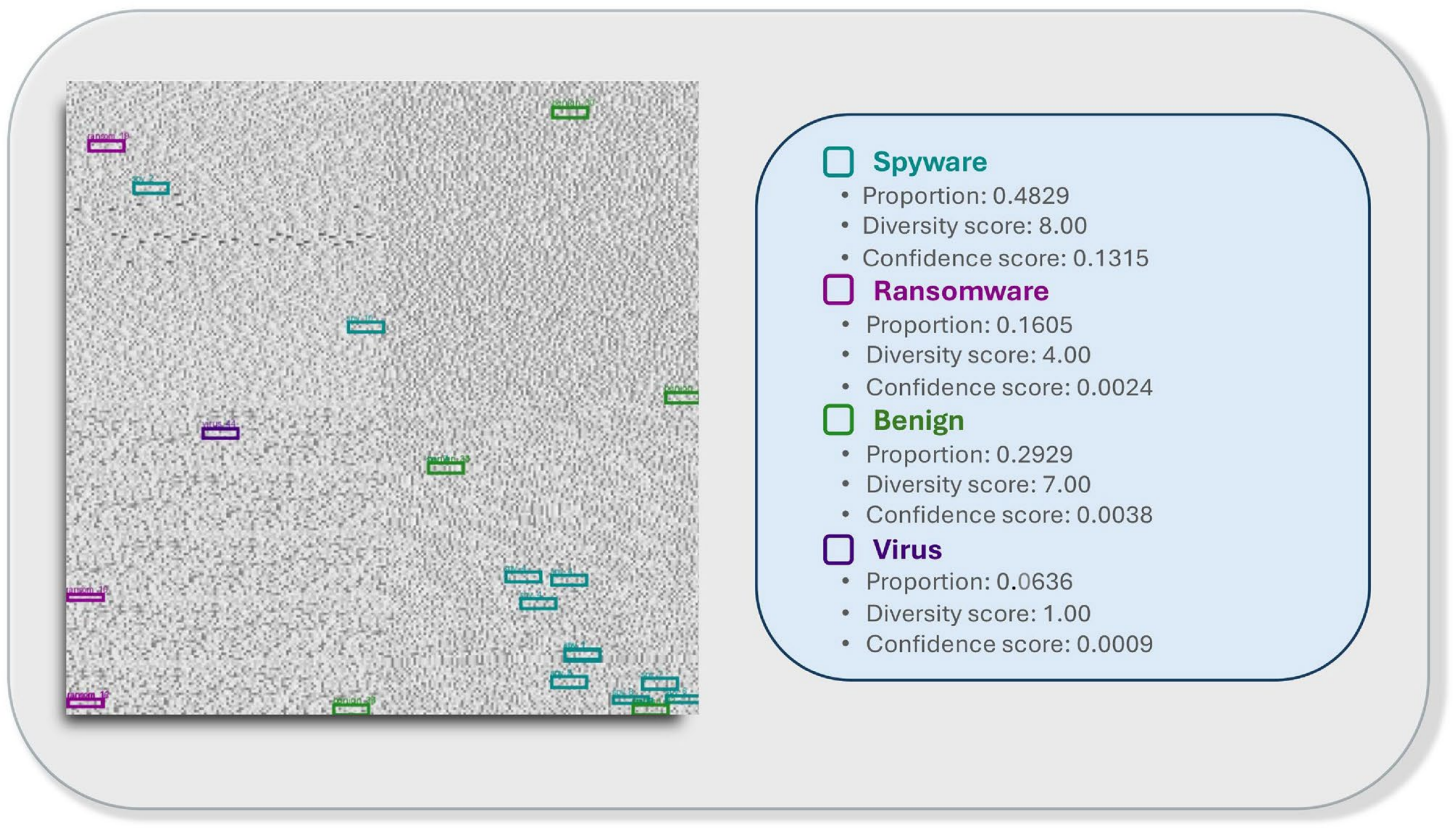
Visualisation of a Spyware sample with bounding boxes, illustrating the percentage distribution of detected behavioural features associated with different malware types.

In the multi-label classification setting, DECODE achieves an accuracy of 85.13%, outperforming InceptionV4, which achieved 81.81% (Table 4). It also demonstrates a significantly lower Hamming Loss (HL) score compared to other models, reflecting fewer incorrect label predictions. These results validate the effectiveness of our multi-label classification approach, offering a more nuanced and comprehensive malware detection framework based on feature-level analysis.

Figure 10 presents an example visualisation of the detection results. The image is classified as spyware but also exhibits features associated with ransomware, benign software, and viruses. Based on the analysis, the sample predominantly demonstrates spyware-like behaviour, accounting for 48.29% of the detected features, represented by eight distinct key feature classes. Additionally, it contains 16.05% ransomware-related features spanning four feature classes, 29.29% benign-related features across seven classes, and 6.36% virus-related features represented by a single feature class. This analysis enables precise localisation of the relevant regions and reveals the underlying behavioural patterns,effectively capturing the “behavioural DNA” of both malware and benign samples.

## Discussion

Building object detection dataset is traditionally performed through manual annotation. This process however is time-consuming and impractical for large-scale datasets. This study minimises human effort by automatically identifying key features through our newly developed Bayesian Grad-CAM to extract salient regions and grouping similar regions to generate object detection annotations. This method is particularly useful for cases where differences between malware types are not visually distinguishable to the human eye. Although this reduces the reliance on manual annotation, it is computationally intensive and time-consuming when processing large volumes of data.

The resulting object detection dataset enables the application of object detection techniques with bounding boxes, allowing the localisation of feature regions associated with different malware types. This facilitates the identification of multiple behavioural traits exhibited by various malware types within a single sample, supporting multi-label analysis. Given the complex and multi-functional nature of malware, single-label classification approaches may lead to misleading conclusions by focusing only on the dominant trait. Our comparative analysis shows that classification performance improves when images are analysed based on the distinct features associated with each malware category. Both multi-label and binary classification show superior performance compared to baseline methods. The proposed approach achieves notably lower HL, along with higher overall accuracy and improved per-class accuracies. In the binary classification setting, it outperforms other methods in terms of accuracy, F1-score, and MCC. While other models achieve lower FNRs, they exhibit significantly higher FPRs. In contrast, DECODE maintains both low FNR (0.0655) and FPR (0.0432), demonstrating a more balanced and effective detection performance. This indicates a stronger capability to accurately detect malware while minimising the misclassification of benign files. Such a balance is crucial for real-world detection scenarios, as it ensures that legitimate files are not mistakenly flagged, while real threats are reliably identified.

To support explainable analysis, we extract the corresponding API call features from the detected key feature regions to provide detailed behavioural interpretations. This level of interpretability helps analysts understand how malware operates and assess the potential impact. Furthermore, DECODE offers a flexible framework for adapting to evolving malware, which may exhibit behaviours beyond those traditionally associated with its category. It learns the key features of malware categories and is thus capable of addressing real-world variants. Even when new variants are released, they often exhibit behavioural similarities and share intent with known malware categories. By identifying these behaviours, we can detect malicious activity. However, to ensure continued effectiveness and generalisability, the dataset must be regularly updated with more diverse behavioural patterns. Achieving this requires access to a large volume of data encompassing a wide range of malware families and behavioural variations.

To further improve the proposed approach, incremental or continual learning can be applied to adapt the model and dataset to emerging malware behaviours. This would not only enrich the dataset with greater variance but also capture previously unidentified patterns within each malware category. To enhance the explanation of malware behaviour in its final stages and provide more detailed insights into its intent, supplementary contextual information could be incorporated into the dataset. This would help address the dual-use nature of certain APIs, as some of these are also commonly used by legitimate software. By incorporating supporting data such as API call arguments, parent-child process relationships, network destinations, and execution context, the model’s ability to confidently explain the intent behind behaviours, and to distinguish between legitimate and malicious activity, can be significantly enhanced. Future work will also establish objective evaluation metrics in collaboration with domain experts, including structured user studies and task-based evaluations assessing accuracy, efficiency, and error reduction. Correctness will be benchmarked against expert-annotated datasets to ensure reliable assessment of explanation quality.

In real-world malware detection scenarios, the proposed DECODE framework offers several practical advantages. By automating the generation of object detection datasets and enabling multi-label classification with explainable visual insights, it reduces the dependency on manual labour while significantly enhancing detection depth and accuracy. This is particularly critical in enterprise and national security environments where rapid and reliable identification of emerging threats is essential. The integration of interpretability not only aids cybersecurity analysts in understanding the reasoning behind detection outcomes but also supports actionable decision-making in threat response and mitigation. Furthermore, DECODE’s ability to detect and explain behavioural patterns in novel or obfuscated malware variants makes it a valuable tool for proactive threat hunting. Its balanced performance, minimising false positives and false negatives, helps maintain system integrity while avoiding disruption to legitimate operations, a key requirement in high-stakes, real-time cybersecurity environments.

## Conclusion

We strongly believe that this study advances beyond traditional malware detection by introducing a novel visual and explainable framework that dissects and interprets the behavioural traits of malware with greater granularity. By highlighting distinctive features of various malware types and localising them within visual representations, this approach empowers analysts to detect, understand, and respond to threats with enhanced precision. Such capabilities are crucial for strengthening cybersecurity resilience in an environment where cyber threats continue to evolve in complexity and scale.

This study addresses key limitations of conventional detection practices through this seminal multi-label classification strategy, which outperforms single-label models in both binary and multi-label contexts. The ability to localise salient feature regions and map them back to API call sequences enables a transparent and interpretable detection process. Further enhancing interpretability, we integrate a multi-agent module that explains these features and their associated labels, transforming complex technical outputs into human-readable insights. Together, these components establish a fully visual and explainable malware detection pipeline, bridging the gap between raw data and actionable intelligence.

Another significant contribution of this work is the introduction of a Bayesian modification of Grad-CAM, which generates uncertainty-aware heatmaps for more reliable visual explanations. We also present the first object detection dataset for malware, enabling the application of advanced object detection techniques in cybersecurity for the first time. Beyond malware analysis, this framework holds potential for broader use cases requiring automated, interpretable dataset generation tailored to complex classification challenges.

By reducing manual annotation requirements, improving interpretability, and maintaining strong classification performance, DECODE offers substantial real-world value. Its balanced detection metrics and ability to generalise to unseen malware behaviours make it particularly well-suited for practical deployment in enterprise and research settings. Ultimately, this work lays a foundation for scalable, explainable, and adaptive threat detection systems that can keep pace with the dynamic threat landscape.

## Data Availability

Data that support the findings of this study have been deposited in https://github.com/dtuuba/DECODE-DEep_Classification_Of_Dynamic_Exploits.

## Code availability

The source code for DECODE is available at: https://github.com/dtuuba/DECODE-DEep_Classification_Of_Dynamic_Exploits.
